# Structural characterization of two solute-binding proteins for *N,N′*-diacetylchitobiose/*N,N′,N′′*-triacetylchitotoriose of the gram-positive bacterium, *Paenibacillus* sp. str. FPU-7

**DOI:** 10.1016/j.yjsbx.2021.100049

**Published:** 2021-06-10

**Authors:** Takafumi Itoh, Misaki Yaguchi, Akari Nakaichi, Moe Yoda, Takao Hibi, Hisashi Kimoto

**Affiliations:** Department of Bioscience and Biotechnology, Fukui Prefectural University, 4-1-1 Matsuokakenjyoujima, Eiheiji-cho, Yoshida-gun, Fukui 910-1195, Japan

**Keywords:** a.a., amino acid, ABC, ATP-binding cassette, DSF, differential scanning fluorimetry, GlcN, D-glucosamine, GlcNAc, *N*-acetyl-D-glucosamine, GH, glycoside hydrolase, OD600, optical density at 600 nm, PDB, Protein Data Bank, PTS, phosphoenolpyruvate phosphotransferase system, r.m.s.d., root mean-square deviation, RU, response unit, SBP, solute binding protein, Se-Met, selenomethionine, TMD, transmembrane domain, ABC transporter, Chitin oligosaccharide, *Paenibacillus*, Solute binding protein, Two-component regulatory system

## Abstract

The chitinolytic bacterium *Paenibacillus* sp. str. FPU-7 efficiently degrades chitin into oligosaccharides such as *N*-acetyl-D-glucosamine (GlcNAc) and disaccharides (GlcNAc)_2_ through multiple secretory chitinases. Transport of these oligosaccharides by *P*. str. FPU-7 has not yet been clarified. In this study, we identified *nagB1*, predicted to encode a sugar solute-binding protein (SBP), which is a component of the ABC transport system. However, the genes next to *nagB1* were predicted to encode two-component regulatory system proteins rather than transmembrane domains (TMDs). We also identified *nagB2*, which is highly homologous to *nagB1*. Adjacent to *nagB2*, two genes were predicted to encode TMDs. Binding experiments of the recombinant NagB1 and NagB2 to several oligosaccharides using differential scanning fluorimetry and surface plasmon resonance confirmed that both proteins are SBPs of (GlcNAc)_2_ and (GlcNAc)_3_. We determined their crystal structures complexed with and without chitin oligosaccharides at a resolution of 1.2 to 2.0 Å. The structures shared typical SBP structural folds and were classified as subcluster D-I. Large domain motions were observed in the structures, suggesting that they were induced by ligand binding via the “Venus flytrap” mechanism. These structures also revealed chitin oligosaccharide recognition mechanisms. In conclusion, our study provides insight into the recognition and transport of chitin oligosaccharides in bacteria.

## Highlights

• *Paenibacillus* sp. str. FPU-7 degrades chitin extracellularly into disaccharides.
• Two genes, *nagB1* and *nagB2*, were predicted to be components of the sugar transporter.
• NagB1 and NagB2 bound to disaccharides and trisaccharides of chitin.
• NagB1 and NagB2 crystal structures were determined at a resolution of 1.2 to 2.0 Å.
• Structures complexed with oligosaccharides reveal their recognition mechanisms.

## Graphical abstract

**Figure:**
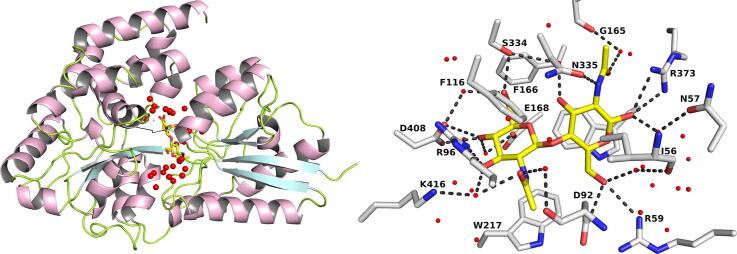

## Introduction

Chitin is produced by arthropods and fungi as a polysaccharide for the cytoskeleton and it is a linear polymer of *N*-acetyl-D-glucosamine (GlcNAc) linked by β-1,4 glycosidic bonds (Aam et al., 2010). Polysaccharides are not only an important source of carbon and nitrogen in marine and terrestrial ecosystems, but are also an important natural resource for the production of a variety of chemicals, and their degradation products, chitin oligosaccharides, are used in several biomedical applications (Kato et al., 2003). The biodegradation of chitin has been explored using various chitinolytic bacteria such as *Serratia marcescens* (Fuchs et al., 1986), *Bacillus circulans* WL-12 (Watanabe et al., 1990), *Paenibacillus* sp. (Itoh et al., 2013), *Streptomyces coelicolor* A3 (Saito et al., 1999), and *Vibrio harveyi* (Montgomery and Kirchman, 1993). The chitin-degrading enzymes of these organisms have also been well studied (Bhattacharya et al., 2007, Dahiya et al., 2006, Eijsink et al., 2010). The glycoside hydrolases (GHs) that act on chitin can have an *endo*-mode of action (endochitinases) or an *exo*-mode of action (exochitinases), and these enzymes act synergistically to release oligosaccharides from insoluble chitin. In addition to GHs, lytic polysaccharide monooxygenases oxidatively cleave chitin (Vaaje-Kolstad et al., 2013). The degraded oligosaccharides are then imported through the bacterial cell membranes, and in the bacterial cytosol or periplasmic space, oligomers are hydrolyzed to GlcNAc by *N*-acetyl-D-glucosaminidases (GlcNAcases) (Itoh and Kimoto, 2019, Vaaje-Kolstad et al., 2013). The obtained GlcNAc is then transformed into the common metabolic intermediate, fructose-6-phosphate, to be further metabolized via glycolysis in the cytosol (Nothaft et al., 2003).

Although a number of chitin-degrading bacteria have been studied, as mentioned above, the only known bacterial transport systems for chitin degradation products, GlcNAc and (GlcNAc)_2_, are those identified in *Streptomyces* sp. and Vibrionaceae. In bacteria, phosphoenolpyruvate phosphotransferase systems (PTS) are utilized to import monosaccharides while ATP-binding cassette (ABC) transporters import di-, tri-, and oligosaccharides. In *S. olivaceoviridis* and *S. coelicolor* A3(2), GlcNAc uptake is mainly achieved by the PTS and ABC transporter NgcEFG (Saito and Schrempf, 2004, Wang et al., 2002, Xiao et al., 2002), which also transports (GlcNAc)_2_. In *S. coelicolor* A3(2), the ABC transporter DasABC transports (GlcNAc)_2_, which is hydrolyzed by the GlcNAcase DasD in the cytosol (Saito et al., 2007). The gram-negative cholera pathogen *Vibrio cholerae* can also use chitin as a source of nutrients. The chitin oligosaccharides produced by the chitinases of *V. cholerae* are transported across the outer membrane via chitoporin to the periplasm, where the saccharides are further broken down into GlcNAc and (GlcNAc)_2_. The resulting monomers and dimers are then transported across the inner membrane by specific transporters, such as PTS and ABC transporters, respectively (Hayes et al., 2017, Hunt et al., 2008, Meibom et al., 2004). The bacterial ABC transporters for uptake (ABC importers) consist of five proteins: a solute binding protein (SBP), two transmembrane domains (TMDs), and two nucleotide-binding domains (ATPases). The free energy of ATP hydrolysis of ATPases is used to import various molecules across the cell membrane, whereas SBP is responsible for specifically trapping ligands and passing them through the pathway formed by the two TMDs (ter Beek et al., 2014).

Recently, we isolated the gram-positive bacterium *Paenibacillus* sp. str. FPU-7 from the soil as a highly chitinolytic bacterium that can degrade insoluble chitin flakes (Itoh et al., 2013). This bacterium contains at least six extracellular chitinases (ChiA, B, C, D, E, and F), which are catabolically inhibited by GlcNAc, and one cell surface-expressed multi-modular chitinase (ChiW) for efficient chitin degradation (Itoh et al., 2014, Itoh et al., 2013, Itoh et al., 2016). ChiW hydrolyzes chitin into (GlcNAc)_2_ on the cell surface, and the expression of ChiW is induced by (GlcNAc)_2_. *P*. str. FPU-7 can then hydrolyze (GlcNAc)_2_ into GlcNAc via GlcNAcase (PsNagA), a GH3 enzyme found in the cytosol (Itoh et al., 2019). However, the mechanism by which chitin oligosaccharides are transported into *P*. str. FPU-7 cells across the cell membrane remains unclear.

In this study, we aimed to elucidate the full extent of (GlcNAc)_2_ transport in *P*. str. FPU-7 and identified two proteins (NagB1 and NagB2) in the draft genome of *P*. str. FPU-7 as SBPs of chitin oligosaccharides. These proteins are not homologous to the previously known SBPs of (GlcNAc)_2_ in *S. olivaceoviridis*, *S. coelicolor* A3(2), or *V. cholera* (sequence identity < 20%). The amino acid (a.a.) sequences and the properties of NagB1 and NagB2 were also different from the chitin-binding domains (or proteins) that work in concert with chitinolytic enzymes. We also determined the affinities of these SBPs for (GlcNAc)_2_ and (GlcNAc)_3_ using surface plasmon resonance and its recognition mechanisms using the crystal structures of the SBPs in the complex formed with the oligosaccharides. Altogether, the present study reports the biochemical and structural features of the SBPs NagB1 and NagB2 that mediate the capture of (GlcNAc)_2_ and (GlcNAc)_3_ within *Paenibacillus* spp.

## Materials and methods

### Chemicals and reagents

Saccharides (GlcNAc)_2_, (GlcNAc)_3_, and (GlcNAc)_4_ were purchased from Tokyo Chemical Industry (Tokyo, Japan). Other chemicals and reagents were of analytical grade and purchased from FUJIFILM Wako Pure Chemical Corporation (Osaka, Japan) or Sigma-Aldrich (St. Louis, MO, USA), unless otherwise noted.

### Amino acid sequence analysis

Sequence analysis was performed using the Pfam database (El-Gebali et al., 2019), using the basic local alignment search tool (BLAST) (Altschul et al., 1990) and ClustalW (Larkin et al., 2007). The N-terminal signal peptides were predicted using the SignalP 5.0 server (http://www.cbs.dtu.dk/services/SignalP/) (Almagro Armenteros et al., 2019). Protein palmitoylation sites were predicted using the CSS-Palm 4.0 server (http://csspalm.biocuckoo.org/index.php) (Ren et al., 2008).

### Cloning of nagB1 and nagB2 genes from *P*. str. FPU-7

The *nagB1* gene (DDBJ/EMBL/GenBank database accession number: LC622162) was subcloned into the expression vector pET21b (Novagen, Madison, WI, USA). Colony PCR was performed using KOD-Plus-Neo polymerase (Toyobo, Osaka, Japan) and a single colony of *P*. str. FPU-7 as template. Synthetic oligonucleotides with *Nde*I and *Xho*I restriction sites were then added to their termini as forward and reverse primers (Table S1). To express a stable recombinant protein, facilitate purification, and obtain good quality crystals, the primers were designed to add a six-histidine tag (LEHHHHHH) to the C-terminus and delete the N-terminal signal peptide (Met1–Ser21) and 27 a.a. residues (Cys22–Phe48). These residues were selected by comparing them to the disordered region of the crystal structure of NagB2, as described in the results section. In contrast, the *nagB2* gene (DDBJ/EMBL/GenBank database accession number: LC622163) was prepared without the predicted N-terminal signal peptide (Met1 to Ala20) and subcloned into the expression vector pET21b (Novagen) with a six-histidine tag at the C-terminus (LEHHHHHH) (Table S1). The PCR products were ligated into the vectors using the In-Fusion HD Cloning Kit (Takara Bio, Kusatsu, Japan). Successful plasmid construction was confirmed by DNA sequencing using the ABI PRISM 3130xl Genetic Analyzer (Applied Biosystems, Foster City, CA, USA).

### Purification of NagB1 and NagB2 recombinant proteins

The expression vector was transformed into competent *Escherichia coli* BL21(DE3) cells (Novagen) or T7 Express Crystal Cells (New England Biolabs Inc., Ipswich, MA, USA). *E. coli* BL21(DE3) transformants were grown in Luria-Bertani medium (0.5 L) containing 50 μg/mL ampicillin at 37 °C. For the expression of a NagB2 derivative with selenomethionine (Se-Met), *E. coli* T7 Express Crystal was aerobically cultured in minimal medium supplemented with 25 μg/mL of Se-Met. When the optical density of the culture measured at 600 nm (OD600) reached 0.6, the cultures were supplemented with 1.0 mM IPTG, followed by incubation at 20 °C for 20 h. The cultured cells were collected by centrifugation at 6000 *× g* at 4 °C for 5 min. The harvested cells were ultrasonically disrupted in 20 mM sodium phosphate buffer (pH 7.4) and 0.1 mM PMSF. Clear solutions were obtained after centrifugation at 15,000 *× g* and 4 °C for 20 min. The proteins were then fractionated with (NH_4_)_2_SO_4_ from 30% to 70% saturation. The precipitants were dissolved in sodium phosphate buffer containing 20 mM imidazole, and applied to a nickel-immobilized metal affinity column (HisTrap HP 5 mL column; Cytiva, Tokyo, Japan). The column-absorbed proteins were eluted with a linear gradient of imidazole (0.02–0.15 M) in sodium phosphate buffer (50 mL). Ammonium sulfate was added to the solution containing proteins to reach 30% saturation. The proteins were further purified by hydrophobic interaction chromatography using a HiTrap Butyl HP 5 mL column (Cytiva) equilibrated with 20 mM Tris buffer (pH 7.5) and 1.2 M (NH_4_)_2_SO_4_ and eluted with a linear gradient of (NH_4_)_2_SO_4_ (1.2–0 M) in the same buffer. The eluted proteins were dialyzed at 4 °C overnight in 10 mM HEPES buffer (pH 7.4) and used as purified protein sources. Protein concentrations were determined by UV spectrophotometry using the theoretical molar extinction coefficients of 43,890 and 42,400 M^−1^ cm^−1^ for NagB1 and NagB2, respectively, according to the ExPASy ProtParam tool server (http://web.expasy.org/protparam/) (Gasteiger et al., 2003). The purity of the proteins was assessed by 12% SDS-PAGE followed by CBB R-250 staining.

### Differential scanning fluorimetry

The differential scanning fluorimetry (DSF) assay mixtures contained 20 mM Tris (pH 7.5), 0.25 mg/mL protein, and SYPRO Orange Protein Gel Stain dye (5 × final concentration, Thermo Fisher Scientific, Waltham, MA, USA) with or without various saccharides (10 mM GlcNAc, (GlcNAc)_2_, (GlcNAc)_3_, (GlcNAc)_4_, D-glucosamine (GlcN), (GlcN)_2_, glucose, maltose, or mannose) in a total volume of 20 μL. The melting curves (increase in fluorescence) were monitored using a real-time PCR instrument (StepOne, Thermo Fisher Scientific) using an ROX filter. Samples were heated from 25 to 95 °C at a constant rate of 1 °C/min. Apparent melting temperatures (*T*_m, app_) were calculated as an inflection point of the melting curve, assuming a two-state unfolding model, using the Protein Thermal Shift software (*T*_m_D in the software, Thermo Fisher Scientific). Assays were performed in triplicate. Δ*T*_m, app_ values were calculated as the difference between the average *T*_m_D values measured with and without the ligand.

### Surface plasmon resonance (SPR)

The affinities of NagB1 and NagB2 for (GlcNAc)_2_ and (GlcNAc)_3_ were evaluated using the Biacore X100 Plus Package (Cytiva). Both proteins were diluted in 10 mM sodium acetate buffer (pH 5.5) to 0.1 mg/mL. The proteins were covalently immobilized onto CM5 sensor chips using the Amine Coupling Kit (Cytiva) to a level of approximately 3000 response units (RU). Sensorgrams were recorded at 25 °C in 10 mM HEPES (pH 7.4), 0.15 M NaCl, 3 mM EDTA, and 0.005% (v/v) surfactant P20. Assays were performed in the range of 0–10 μM saccharides. The equilibrium dissociation constants (*K*_D_) and other kinetic parameters were calculated by fitting a one-site binding model using the Biacore X100 evaluation software.

### Culture conditions for *P*. str. FPU-7 and analysis of bacterial transportation of (GlcNAc)_2_

To attain an efficient cell yield, *P*. str. FPU-7 was grown at 30 °C with shaking in a medium containing 1.0% (w/v) hipolypepton N and 0.5% (w/v) NaCl at pH 7.5 (hipolypepton N medium) (Itoh et al., 2013). For the (GlcNAc)_2_ transportation analysis, *P*. str. FPU-7 cells were grown in hipolypepton N medium supplemented with 2 mM (GlcNAc)_2_. Aliquots (100 μL) were taken from the medium at appropriate time intervals and centrifuged at 12,000 *× g* for 5 min. The supernatants were filtered using Ultrafee-CL centrifugal filter devices with 0.45 μm pore size membranes (Merck Millipore, Billerica, MA, USA). The filtrated culture media (5 µL) were analyzed using the LC-20AD HPLC system (Shimadzu, Kyoto, Japan) equipped with a TSKgel Amide-80 column (2.0 × 250 mm; Tosoh Co., Tokyo, Japan). (GlcNAc)_2_ was eluted with a mobile phase containing 77% (v/v) acetonitrile and detected at 210 nm wavelength.

### RNA extraction and quantitative real-time PCR (qPCR)

*P*. str. FPU-7 cells were grown at 30 °C with shaking in hipolypepton N medium with or without 2 mM (GlcNAc)_2_. When the OD600 reached 0.5, *P*. str. FPU-7 cells were collected from the culture medium (4 mL) by centrifugation at 6,000 *× g* for 5 min at 4 °C. Total RNA from cultured bacteria was extracted using the FastGene RNA extraction kit (NIPPON Genetics, Tokyo, Japan). Complementary DNA (cDNA) was synthesized by random hexamer priming using the ReverTra Ace qPCR RT Kit (Toyobo). Gene expression was subsequently detected in triplicate using the THUNDERBIRD SYBR qPCR Mix kit (Toyobo) on a qTOWER^3^ G thermal cycler (Analytik Jena AG, Jena, Germany). All quantification data were normalized to the 16S rRNA gene of *P*. str. FPU-7. The primers used are shown in Table S1.

### Immunoblot analysis of NagB1 and NagB2 of *P*. str. FPU-7

Rabbit anti-NagB1 and NagB2 antisera were raised against the recombinant proteins and collected by Eurofins Genomics (Tokyo, Japan). *P*. str. FPU-7 cells were grown at 30 °C with shaking in hipolypepton N medium with or without 2 mM (GlcNAc)_2_. When OD660 reached 0.5, *P*. str. FPU-7 cells were collected from the culture medium (0.5 mL) by centrifugation at 6,000 *× g* for 5 min at 4 °C. The cells were then ultrasonically disrupted in 100 μL of 20 mM sodium phosphate buffer (pH 7.4). The remaining bacterial debris and non-lysed cells were removed by centrifugation at 15,000 *× g* for 10 min at 4 °C. Protein concentrations were determined using the Bio-Rad protein assay kit (Bio-Rad Laboratories, Hercules, CA, USA) with bovine serum albumin as the standard, based on the Bradford method (Bradford, 1976). Protein samples were separated by SDS-PAGE and transferred onto a PVDF membrane (Immobilon-P, PVDF, 0.45 μm, Merck Millipore) by electroblotting. After transfer, the PVDF membrane was blocked with Blocking One (Nacalai Tesque, Inc., Kyoto, Japan) for 1 h. The membrane was washed with 25 mM Tris (pH 7.4), 137 mM NaCl, and 0.05% (v/v) Tween 20. Then, anti-NagB1 or anti-NagB2 (diluted 1:5,000 in Can Get Signal Solution 1, Toyobo) was added, and the blot was incubated for 1 h. Next, affinity isolated alkaline phosphatase-conjugated swine anti-rabbit immunoglobulins (Agilent, Santa Clara, CA, USA) was added at a 1:5000 dilution in Can Get Signal Solution 2 (Toyobo) and incubated for 1 h. Bands were visualized using the BCPIP-NBT solution kit (Nacalai Tesque).

### Crystallization and X-ray diffraction

Purified proteins were concentrated using Amicon Ultra concentrators with a 10,000 molecular weight cutoff membrane (Merck Millipore) to a final concentration of 10 mg/mL. Protein solutions for crystallization were prepared to contain 10 mM (GlcNAc)_2_. Initial screening for the crystallization of NagB1 and NagB2 was performed using commercially available crystallization kits, Crystal Screen 1 and 2 (Hampton Research, Aliso Viejo, CA, USA), and Wizard Classic Crystallization Screens 1 and 2 (Emerald BioSystems, Bedford, MA, USA), with the sitting-drop vapor-diffusion method in 96-well plates at 20 °C. The crystallization conditions were further refined using a 24-well plate and performed using the sitting-drop vapor-diffusion method at 20 °C. The drop (4 μL) consisted of 2 μL of protein solution and 2 μL of reservoir solution (0.5 mL) containing 20% (w/v) PEG4000, 0.2 M sodium acetate, and 0.1 M Tris (pH 8.5) for the crystallization of NagB1 with (GlcNAc)_2_ and 20% (w/v) PEG3350 and 0.1 M MES (pH 6.0) for that of NagB2 with or without (GlcNAc)_2_ and (GlcNAc)_3_.

Single crystals were soaked in crystallization solutions supplemented with 30% (w/v) glycerol for NagB1 or 30% (w/v) PEG400 for NagB2, followed by cooling in a stream of cold nitrogen gas. X-ray diffraction images were acquired for the native and Se-Met derivative crystals at −173 °C under a nitrogen gas stream with a MAR MX 225HS or 225HE detector (Rayonix, L.L.C., Evanston, IL, USA) and synchrotron radiation (λ = 0.85 or 1.0 Å for the native crystal or 0.9790 Å for the Se-Met derivative crystal) at the BL-26B1 or BL-38B1 stations of SPring-8 (Japan) and then integrated and scaled using HKL-2000 (Otwinowski and Minor, 1997) (Table 1).

**Table 1:** Data collection and refinement statistics for NagB1 and NagB2 structures.

	NagB1/(GlcNAc)_2_	NagB2 (Se-Met)	NagB2	NagB2/(GlcNAc)_2_	NagB2/(GlcNAc)_3_
PDB accession number	7EHP		7EHO	7EHQ	7EHU
Diffraction source	BL26B1	BL38B1	BL26B1	BL38B1	BL38B1
Space group	C2	*P*2_1_	*P*2_1_	C2	*P*2_1_
*a*, *b*, *c, β* (Å, °) or *a*, *c* (Å)	196.0, 49.8, 77.9, 107.1	61.6, 76.4, 87.5, 106.5	61.8, 76.0, 87.5, 107.1	136.5, 68.7, 56.8, 101.6	50.2, 69.6, 56.2, 108.4
Resolution limit (Å)^a^	50.0–2.00 (2.03–2.00)	50.0–2.40 (2.44–2.40)	50.0–1.80 (1.83–1.80)	50.0–1.70 (1.73–1.70)	50.0–1.20 (1.22–1.20)
Measured reflections	228,845	221,480	417,923	360,655	842,185
Unique reflections	47,871 (2402)	30,511 (1516)	65,822 (3323)	55,696 (2800)	114,172 (5714)
Completeness (|*I*|>σ|*I*|) (%)	99.8 (100.0)	99.9 (100.0)	91.2 (92.0)	98.0 (98.9)	100.0 (100.0)
Redundancy	4.8 (4.6)	7.3 (7.1)	6.3 (5.7)	6.5 (6.7)	7.4 (7.2)
<*I*/σ(*I*)>	12.7 (2.1)	28.3 (4.6)	20.5 (1.5)	54.8 (2.2)	41.6 (2.2)
*R*_meas_	0.214 (1.098)	0.120 (0.518)	0.124 (1.313)	0.079 (1.288)	0.078 (1.279)
*R*_pim_	0.099 (0.511)	0.045 (0.194)	0.048 (0.533)	0.031 (0.492)	0.029 (0.474)
CC_1/2_ of last shell	0.642	0.896	0.701	0.703	0.702
Wilson *B* factor (Å^2^)	18.1	29.2	17.8	26.8	10.9

Refinement					
Final model	397 a.a. (A), 393 a.a. (B), 633 waters, and two (GlcNAc)_2_		406 a.a. (A), 402 a.a. (B), 916 waters, and tetraethylene glycol	406 a.a., 403 waters, and (GlcNAc)_2_	405 a.a., 639 waters, (GlcNAc)_3_, and diehylene glycol
Resolution limit (Å)	38.93–2.01 (2.05–2.01)		41.81–1.79 (1.82–1.79)	27.89–1.70 (1.73–1.70)	29.15–1.20 (1.21–1.20)
Used reflections	47,843 (2,309)		65,787 (2,512)	55,593 (2,783)	114,124 (3,584)
Completeness (|*F*| > σ|*F*|) (%)	98.7 (82.0)		90.8 (83.0)	97.6 (93.0)	99.8 (96.0)

Average *B*-factor (Å^2^)					
Protein	26.2 (A), 24.8 (B)		19.4 (A), 27.0 (B)	35.8	17.7
Water	30.2		31.0	43.9	29.2
(GlcNAc)_2_ or (GlcNAc)_3_	17.3 (A), 14.0 (B)			26.2	10.9
Tetraethylene glycol or diethylene glycol			30.1		44.8
*R*-factor (%)	17.8 (23.6)		18.0 (28.7)	19.5 (33.9)	14.2 (22.8)
*R*_free_ (%)	22.5 (32.7)		20.7 (33.7)	22.2 (36.8)	16.4 (23.9)
Root-mean-square deviations					
Bond (Å)	0.009		0.010	0.013	0.012
Angle (°)	1.136		1.623	1.864	2.049
Ramachandran plot (%)					
Favored region	96.68		97.64	97.77	98.76
Allowed region	3.32		2.36	2.23	1.24
Outlier region	0		0	0	0

a Data in the highest resolution shells are given in parentheses.

### Structure determination and refinement

Phase determination and initial model building were performed using the processed Se-Met anomalous dataset of NagB2 (Table 1) and the SHELX(CDE) (Sheldrick, 2015) programs implemented in the HKL2MAP program package. The partial model obtained was manually rebuilt using Coot ver. 0.9 (Winn et al., 2011). The model was refined using the Phenix.refine program in the PHENIX package (Adams et al., 2010) against the native NagB2 dataset (Table 1). Several rounds of refinements and manual model building were performed for the 1.8 Å dataset. Water molecules were automatically incorporated where the *F*_o_–*F*_c_ and 2*F*_o_–*F*_c_ electron density maps showed densities greater than 3.0 σ and 1.0 σ, respectively. A number of water molecules were manually identified based on the density maps. The crystal structures of NagB1 and NagB2 complexed with (GlcNAc)_2_ or (GlcNAc)_3_ were determined by the molecular replacement method using the Phaser program (McCoy et al., 2007) in the PHENIX package, employing the structure of the oligosaccharide-unbound NagB2 structure as a search model. The oligosaccharide-bound structures were refined and manually rebuilt using Phenix.refine and Coot (Table 1). Initially, GlcNAc residues were manually placed and linked with β-1,4-linkages based on the interpretation of electron density shapes in the 2*F*_o_-*F*_c_ and *F*_o_-*F*_c_ maps. Several rounds of refinement were conducted, followed by manual model building to improve the model. Structural similarity was screened using the Protein Data Bank (PDB) (Berman et al., 2000) and the DALI program (Dietmann et al., 2001). Large-scale hinge bending motions were analyzed using the DynDom web server (Hayward and Lee, 2002). Structural alignments were conducted by superimposition using a secondary structure matching program (Krissinel and Henrick, 2004) in Coot. The conservation of the a.a. sequence on the surface of the structure was estimated using the ConSurf server (Ashkenazy et al., 2016). The server performed a three-iteration CSI-BLAST (Angermüller et al., 2012) search and selected 150 homologous sequences (E-value score, 8.9 × 10^−222^ to 3.9 × 10^−84^) from the UniRef90 database (Suzek et al., 2015) for multiple sequence alignment. The electrostatic potential was calculated using APBS and PDB2PQR (Unni et al., 2011). Structural figures were prepared using PyMol 2.5 (Schrödinger, New York, NY, USA).

### Accession numbers

The nucleotide sequence data of *nagB1* and *nagB2* are available in the DDBJ/EMBL/GenBank databases under the accession numbers LC622162 (*nagB1*) and LC622163 (*nagB2*). The coordinates and structure factors of NagB1 and NagB2 are available in the PDB under the accession numbers 7EHP (NagB1/(GlcNAc)_2_), 7EHO (NagB2), 7EHQ (NagB2/(GlcNAc)_2_), and 7EHU (NagB2/(GlcNAc)_3_).

## Results

### Production and purification of recombinant NagB1 and NagB2

**Fig. 1:**
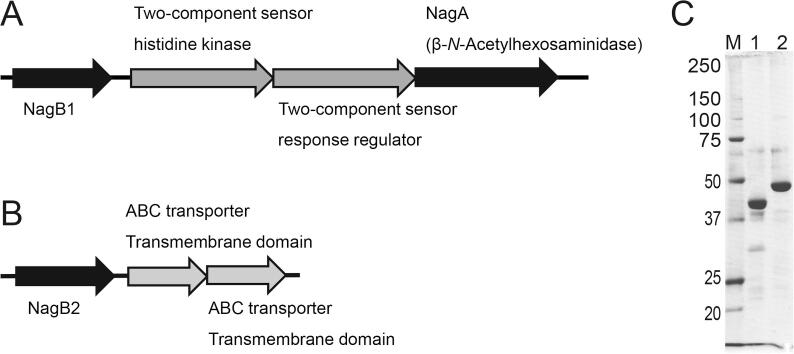
Genome contexts of *nagB1* and *nagB2* and SDS-PAGE profile of the recombinant proteins. (A) In the draft genome of *P*. str. FPU-7, *nagB1* was found in the neighborhood of *nagA*, which encodes GlcNAcase. NagB1 was predicted to be a saccharide solute-binding protein. The two genes adjacent to *nagB1* were predicted to encode a two-component regulatory sensor histidine kinase and a response regulator. (B) The *nagB2* gene, which is homologous to *nagB1*, was also found in the genome. The two genes adjacent to *nagB2* were predicted to encode transmembrane domains (TMDs) of ABC transporter proteins. (C) NagB1 and NagB2 were prepared as recombinant proteins in *E. coli*. Protein bands were stained with CBB R-250. Lane M, molecular mass standards (kDa); lane 1, partially purified recombinant NagB1; lane 2, partially purified recombinant NagB2.

The bacterium *P*. str. FPU-7 can degrade chitin to (GlcNAc)_2_ in the culture medium (Itoh et al., 2013). Recently, we characterized GH3 GlcNAcase (PsNagA), which hydrolyzes (GlcNAc)_2_ to GlcNAc in the cytosol of *P*. str. FPU-7 (Itoh et al., 2019). In the present study, we re-analyzed the draft genome and open reading frames of *P*. str. FPU-7 using BLAST. In the sequence near *nagA*, we found a 1,197-bp gene (denoted as *nagB1*), which was the most promising candidate for a chitin oligosaccharide-binding protein on the cell membrane able to import the saccharides (Fig. 1A). A gene homologous to *nagB1* (*nagB2*) was also found in the draft genome (Fig. 1B), and their a.a. sequences were highly similar (a.a. sequence identity = 46.4%) (Supplementary Fig. S1). Both a.a. sequences showed identity with those of other SBPs for saccharides such as the xylooligosaccharide-binding protein XBP1 of *Caldanaerobius polysaccharolyticus* (identity = 27% for NagB1 and 28% for NagB2) (Han et al., 2012) and the arabinoxylooligosaccharide-binding protein BIAXBP of *Bifidobacterium animalis* subsp. *Lactis* Bl-04 (identity = 26% for NagB1 and 27% for NagB2) (Ejby et al., 2013). Signal peptide sequences were predicted at their N-terminal regions (21 a.a. residues, Met1–Ser21 of NagB1 and Met1–Gly21 of NagB2), and the palmitoylation sites were also predicted at Cys22 for both NagB1 and NagB2. Both proteins were expected to be anchored to the plasma membrane by palmitic acid.

Recombinant proteins were produced using an *E. coli* expression system. To obtain stable protein and good quality crystals, recombinant NagB1 protein without the predicted N-terminal signal peptide (Met1 to Ser21) and 27 a.a. residues (Cys22 to Phe48) was prepared by genetic engineering. The 27 deleted residues corresponded to the disordered region of the crystal structure of NagB2, as described below. NagB2 was prepared without the signal peptide (Met1–Ala20). Approximately 40–50 mg of each recombinant protein was purified from to 3–4 g *of E. coli* cells (Fig. 1C).

### DSF analysis of NagB1 and NagB2 with several saccharides

**Fig. 2:**
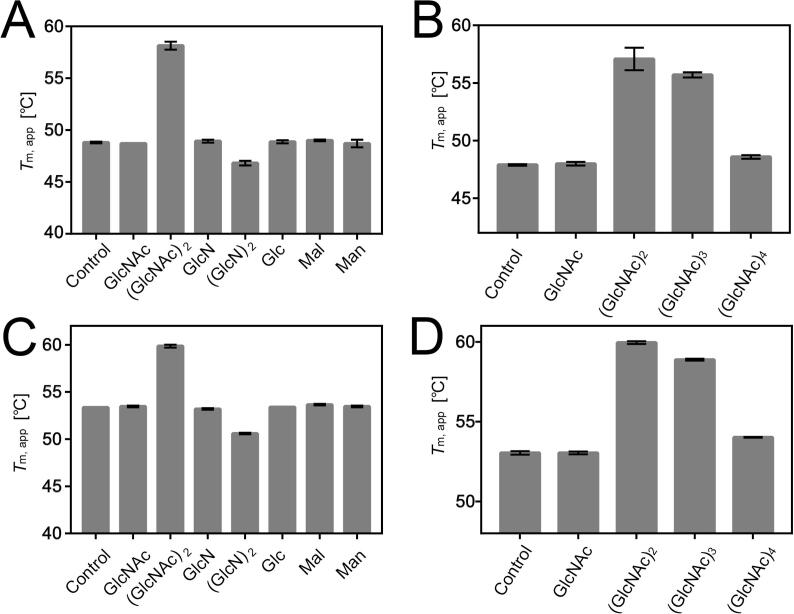
DSF analysis of NagB1 and NagB2 saccharide binding profiles. Average *ΔT*_m, app_ values in the presence of 10 mM of each saccharide are shown. Bars represent standard deviation (n = 3). (A) NagB1 was stabilized by the addition of (GlcNAc)_2_, but not by other saccharides such as GlcNAc, GlcN, (GlcN)_2_, Glc, Mal, and Man. (B) NagB1 was stabilized by the addition of (GlcNAc)_2_ and (GlcNAc)_3_, but not by other chitin oligosaccharides. (C, D) NagB2 was stabilized by (GlcNAc)_2_ and (GlcNAc)_3_ only, as was NagB1.

To determine whether NagB1 and NagB2 could bind to chitin oligosaccharides, DSF assays were performed with chitin oligosaccharides or other saccharides (Fig. 2). The fluorescence of SYPRO Orange, which binds upon denaturation, was measured during heat treatment (25–95 °C). In all experiments, similar melting curve shapes were observed, and the apparent melting temperature (*T*_m, app_) of the proteins was determined as the midpoint of the increase in fluorescence intensity, assuming a two-state unfolding model. The addition of 10 mM (GlcNAc)_2_ and (GlcNAc)_3_ markedly increased the thermal stability of NagB1 and NagB2 with Δ*T*_m, app_ values of 5.7 °C to 9.2 °C, respectively. In contrast, other saccharides (also at 10 mM), such as GlcNAc, (GlcNAc)_4_, GlcN, (GlcN)_2_, glucose, mannose, and maltose, yielded Δ*T*_m, app_ of −2.0 to 0.2 °C. These results suggested that NagB1 and NagB2 are thermally stabilized because of their specific binding to (GlcNAc)_2_ and (GlcNAc)_3_.

### Affinity of NagB1 and NagB2 for (GlcNAc)_2_ and (GlcNAc)_3_

**Fig. 3:**
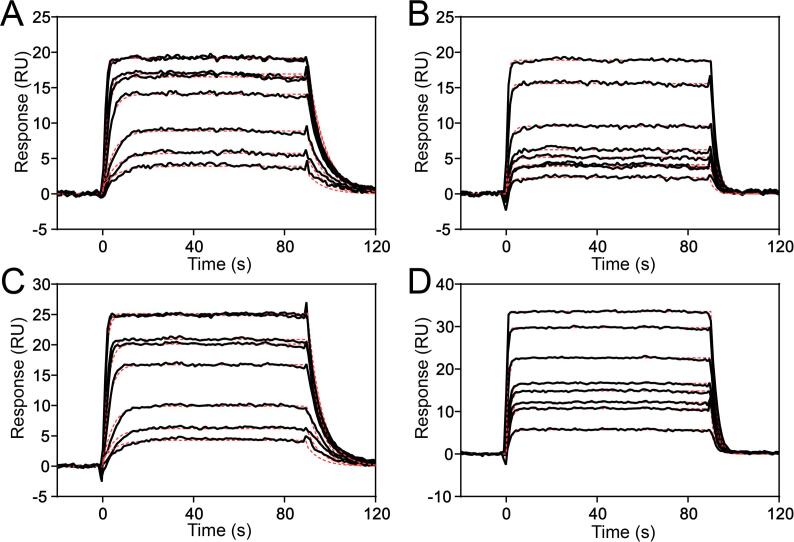
Binding sensorgrams of NagB1 and NagB2. Both proteins were captured onto the surface of CM5 chips using an Amine Coupling Kit. Various concentrations of oligosaccharides were injected across the surface, and binding was monitored using surface plasmon resonance. (A) Sensorgram of the interaction between NagB1 and (GlcNAc)_2_ (from bottom to top, 0.025, 0.5, 0.1, 0.25, 0.4, 0.5, 0.8, and 1 µM (GlcNAc)_2_). (B) Sensorgram of the interaction between NagB1 and (GlcNAc)_3_ (from bottom to top, 0.2, 0.4, 0.6, 0.8, 1, 2, 5, and 10 µM (GlcNAc)_3_). (C) Sensorgram of the interaction between NagB2 and (GlcNAc)_2_ (from bottom to top, 0.025, 0.5, 0.1, 0.25, 0.4, 0.5, 0.8, and 1 µM (GlcNAc)_2_). (D) Sensorgram of the interaction between NagB2 and (GlcNAc)_3_ (from bottom to top, 0.2, 0.4, 0.6, 0.8, 1, 2, 5, and 10 µM (GlcNAc)_3_). The kinetic parameters and dissociation constants obtained from these curves are listed in Table 2.

The binding kinetics of NagB1 and NagB2 for (GlcNAc)_2_ and (GlcNAc)_3_ were analyzed using SPR (Fig. 3, Table 2). The dissociation constants (*K*_D_) were on the order of 10^-7^–10^-6^ M, which is the same range as those of other sugar SBPs (Berntsson et al., 2010), and the binding of (GlcNAc)_2_ on NagB1 showed the highest affinity (*K*_D_ = 1.73 × 10^-7^ M) (Table 2). The *K*_D_ values of both proteins for (GlcNAc)_2_ were higher than those for (GlcNAc)_3_, owing to faster association rate constants (*k*_a_) and slower dissociation rate constants (*k*_d_). Although the affinities of both proteins for (GlcNAc)_2_ were comparable, NagB2 had a higher affinity for (GlcNAc)_3_ than NagB1. The *k*_d_ of NagB2 for (GlcNAc)_3_ was similar to that of NagB1 for (GlcNAc)_3_. However, the *k*_a_ of NagB2 for (GlcNAc)_3_ was higher than that of NagB1 for (GlcNAc)_3_.

**Table 2:** Kinetics of chitin oligosaccharide binding to NagB1 and NagB2.

Protein	Ligand	*k*_a_ × 10^5^ (M^−1^ s^−1^)	*k*_d_ (s^−1^)	*K*_D_ (μM)	χ*^2^*^a^
NagB1	(GlcNAc)_2_	7.81 ± 0.11	0.135 ± 0.001	0.173	0.112
	(GlcNAc)_3_	1.40 ± 0.05	0.414 ± 0.008	2.97	0.135
NagB2	(GlcNAc)_2_	7.91 ± 0.11	0.150 ± 0.001	0.190	0.120
	(GlcNAc)_3_	3.83 ± 0.08	0.428 ± 0.004	1.12	0.162

a Averaged squared residual per data point (χ^2^ = Σ (*y*_i_-*ŷ*_i_)^2^/(*n*-*p*)), where *y*_i_ is the fitted value at each data point, *ŷ*_i_ is the experimental value at the point, *n* is the number of data points, and *p* is the number of fitted parameters.

### Expression of NagB1 and NagB2 in *P*. str. FPU-7

**Fig. 4:**
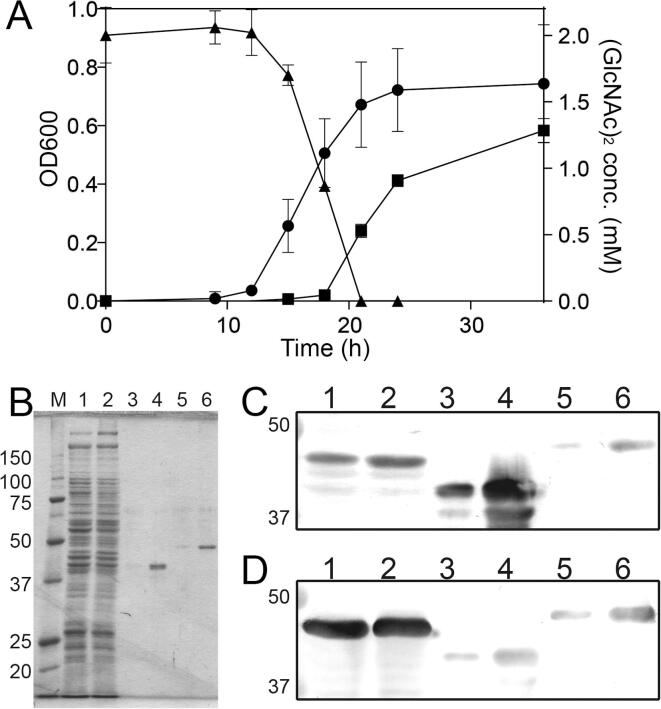
Protein expression profiles of NagB1 and NagB2. (A) *P*. str. FPU-7 grew faster in media supplemented with 2 mM (GlcNAc)_2_ (black circles) than in media without (GlcNAc)_2_ (black squares). The concentration of (GlcNAc)_2_ in the medium (black triangles) decreased with the growth of *P*. str. FPU-7. (B) Cell extracts were visualized by SDS-PAGE. Protein bands were stained with CBB R-250. Lane M, molecular mass standards (kDa); lane 1, cell extracts (5 µg total protein) of *P*. str. FPU-7 grown in the medium without (GlcNAc)_2_; lane 2, cell extracts (5 µg total protein) of *P*. str. FPU-7 grown in the medium supplemented with 2 mM (GlcNAc)_2_; lane 3, partially purified recombinant NagB1 (10 ng total protein); lane 4, partially purified recombinant NagB1 (100 ng total protein); lane 4, partially purified recombinant NagB2 (10 ng total protein); lane 5, partially purified recombinant NagB2 (100 ng total protein). (C, D) Proteins separated on SDS-PAGE gels were transferred to PVDF membranes and then incubated with antisera of recombinant NagB1 (C) or NagB2 (D). The contents of each lane were the same as in SDS-PAGE in Fig. 4B. Although both antibodies reacted with both recombinant proteins, at least one of them was expressed with (Fig. 4B–D, lane 1) or without the addition of (GlcNAc)_2_ (Fig. 4B–D, lane 2).

*P*. str. FPU-7 is unable to grow well in minimal media such as M9 (Itoh et al., 2013). Thus, the bacterium was cultured in a medium containing 1.0% (w/v) hipolypepton N and 0.5% (w/v) NaCl supplemented with or without 2 mM (GlcNAc)_2_. Hipolypepton N is made from soybean hydrolysate and it is considered almost free of (GlcNAc)_2_. Bacterial growth was slightly faster in the (GlcNAc)_2_-containing medium than in the medium without disaccharide. As the bacteria grew, the disaccharide in the medium was consumed and completely disappeared after 21 h (Fig. 4A). The expression of each gene was similar in the presence and absence of (GlcNAc)_2_. In the medium without (GlcNAc)_2_, the expression of *nagB1* in the medium without (GlcNAc)_2_ was 0.73 ± 0.13 fold and that of *nagB2* was 0.42 ± 0.20 fold compared with that in the medium with (GlcNAc)_2_. Bacterial cell extracts were then immunoblotted with each antibody to confirm whether *P*. str. FPU-7 produced NagB1 and NagB2 proteins (Fig. 4B–D). Unfortunately, both antibodies reacted against both proteins (Fig. 4C, D, lanes 3–6), and it was not possible to identify whether only one or both proteins were produced. However, at least one protein was produced in sufficient amount with the addition of (GlcNAc)_2_ (Fig. 4C, D, lane 1) or without it (Fig. 4C, D, lane 2). When 5 μg of protein from the cell extract was loaded per lane (Fig. 4B-D, lanes 1 and 2), the presence of more than 100 ng of the binding protein was confirmed (Fig. 4C, D, lanes 3 and 5).

### Overall structure of NagB1

**Fig. 5:**
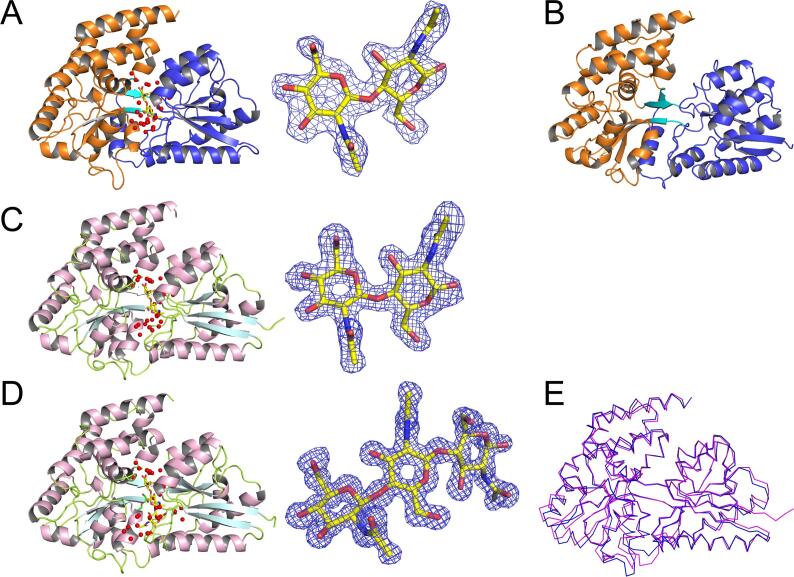
Overall structures of NagB1 and NagB2. The structures are represented by ribbon models: NagB1/(GlcNAc)_2_ (A), NagB2 (B), NagB2/(GlcNAc)_2_ (C), and NagB2/(GlcNAc)_3_ (D). The electron density around (GlcNAc)_2_ or (GlcNAc)_3_ was depicted as a composite omit map (2m*F*_o_-D*F*_c_) with simulated annealing and contoured at the 1.0 σ level. The structures of NagB1 (A) and NagB2 (B) adopt a typical SBP-fold consisting of two globular α/β domains, N-terminal domain I (blue) and C-terminal domain II (orange ribbon model), and three hinge regions made up of antiparallel β-strands between the two domains (cyan). (GlcNAc)_2_ (A, C) or (GlcNAc)_3_ (D) (yellow stick model) were bound to the ligand-binding site, along with many water molecules (red balls). (E) The main chain structures of NagB1/(GlcNAc)_2_ (blue) and NagB2/(GlcNAc)_2_ (magenta) are very similar with r.m.s.d. of 1.0 Å for the 394 Cα atoms. (For interpretation of the references to colour in this figure legend, the reader is referred to the web version of this article.)

The crystal structure of NagB1 in complex with (GlcNAc)_2_ (NagB1/(GlcNAc)_2_) was determined at 2.0 Å (Fig. 5A, Table 1). To date, no ligand-free form of NagB1 has been obtained. The asymmetric unit of NagB1/(GlcNAc)_2_ contains two polypeptide chains (A and B). There were no disordered regions, except for the nine C-terminal residues, including the *hexa*-histidine tag of both chains and four residues (Asp299–Lys302) of chain B. The two polypeptide chains were virtually identical and formed the same folded structure with a root mean square deviation (r.m.s.d.) of 0.57 Å for all Cα atoms. The overall structure of NagB1 adopts a typical SBP-fold (Berntsson et al., 2010) consisting of two globular α/β domains: N-terminal domain I (residues Thr49–Thr162 and Ser330–Arg395; Fig. 5A blue ribbon model) and C-terminal domain II (residues Glu167–Met324 and Ala399–Lys445; Fig. 5A orange ribbon model) and three hinge regions composed of antiparallel β-strands between the two domains (residues Ala163–Val166, Ile325–Tyr329, and Gln396–Pro398; Fig. 5A cyan ribbon model). Domain I contains five β-strands and seven α-helices, whereas domain II contains five β-strands and 12 α-helices. The ligand-binding site is buried at the interface between domains.

The DALI server indicated that the NagB1/(GlcNAc)_2_ structure was similar to those of various sugar SBPs such as the glucose binding protein (PDB: 5DVI) of *Pseudomonas putida* CSV86 (Pandey et al., 2016), with a Z-score of 40.3 (r.m.s.d. for the 396 Cα atoms = 2.5 Å and an a.a. sequence identity = 19%), xylooligosaccharide-binding protein BxlE (PDB: 3VXB) from *S. thermoviolaceus* OPC-520 (Tomoo et al., 2017) with a Z-score of 38.3 (r.m.s.d. = 4.3 Å (399 atoms) and a.a. sequence identity = 24%), and raffinose binding protein RafE (PDB: 6PRE) of *Streptococcus pneumoniae* (Hobbs et al., 2019) with a Z-score of 38.0 (r.m.s.d. = 2.6 Å (386 atoms) and a.a. sequence identity = 20%). Although these sugar SBPs have related structures, they share low sequence identities and have different sugar-binding specificities.

### Overall structure of NagB2

The crystal structures of NagB2 in ligand-free or complex forms with (GlcNAc)_2_ (NagB2/(GlcNAc)_2_) and (GlcNAc)_3_ (NagB2/(GlcNAc)_3_) were determined at 1.2 to 1.8 Å resolution (Fig. 5B–D, Table 1). Although their crystallization conditions were the same, the space groups and unit cell parameters differed. The N-terminal a.a. residues were disordered and could not be modeled. There were two polypeptide chains in the asymmetric unit of the crystal of the ligand-free form (chain A, Asp47–Leu452 and chain B, Val49–Gly450), whereas there was only one polypeptide chain in the ligand-bound form. As indicated by the a.a. sequence similarity, the overall structure of NagB2 was very similar to that of NagB1, with an r.m.s.d. of 1.0 Å for the 394 Cα atoms between the (GlcNAc)_2_ bound forms (Fig. 5E). Similar hits to those of NagB1 were obtained for the structural homology search of NagB2 by DALI. The structures of NagB2 were similar to those of the glucose-binding protein (PDB: 5DVI) (Pandey et al., 2016), β-D-galactopyranose-binding protein AcbH of *Actinoplanes* sp. SE50/110 (PDB: 3OO9) (Licht et al., 2011), and BxlE (PDB: 3VXB) (Tomoo et al., 2017).

### Architecture of the ligand binding sites of NagB1 and NagB2

**Fig. 6:**
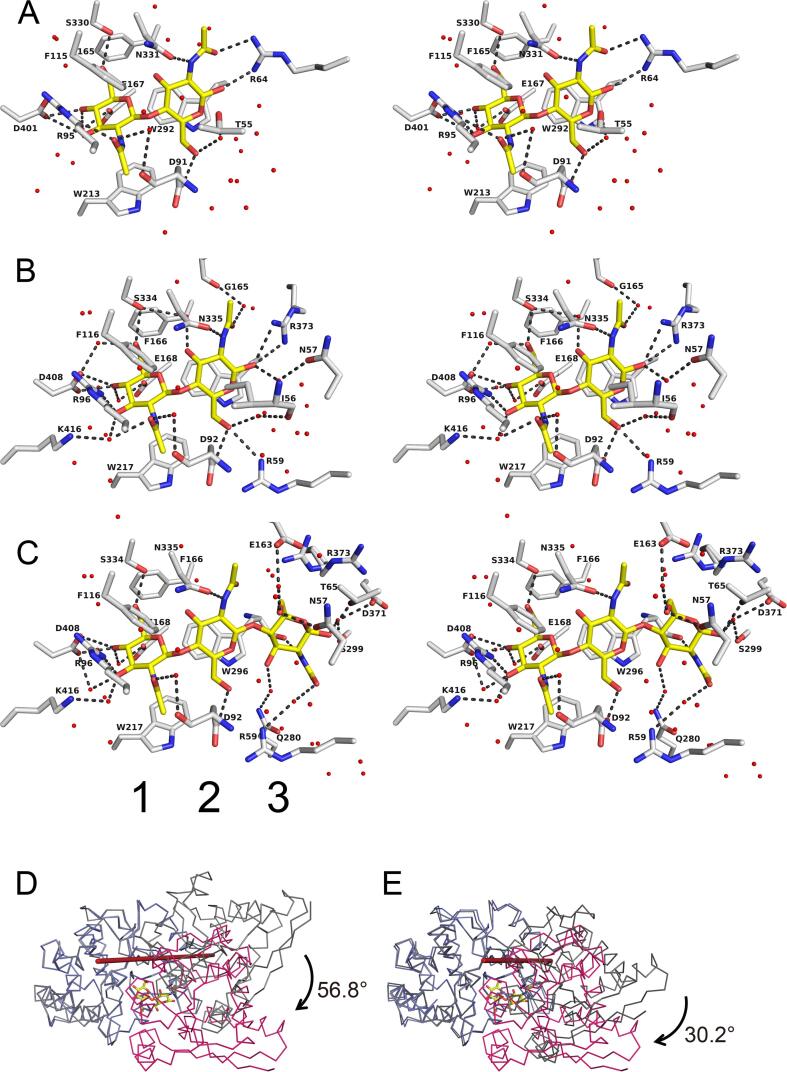
Oligosaccharide binding of NagB1 and NagB2. Close-up view of the ligand-binding sites of NagB1 (A) and NagB2 (B, C). (A-C) The a.a. residues (represented by gray stick models) and water molecules (red balls) interacting with (GlcNAc)_2_ (A, B) or (GlcNAc)_3_ (C) (yellow stick model) are shown. The hydrogen bonds are represented by the black dashed lines. Distances between the atoms are within 3.5 Å. Numbers 1, 2, and 3 indicate the number of saccharide locations from the non-reducing saccharide. (D, E) Upon ligand binding, a conformational change occurs through rigid body rotation, similar to other SBPs. The rotation axis is represented by a red line. In ligand-binding structures, these two domains form closed structures. In the asymmetric unit of the ligand-free-form crystal of NagB2, the domain opening structures were different between the two polypeptide chains (open and semi-closed). The angle of rotation during ligand binding was 56.8° for (GlcNAc)_2_-closed and -open (D) and 30.2° for (GlcNAc)_2_-closed and -semi-closed (E). (For interpretation of the references to colour in this figure legend, the reader is referred to the web version of this article.)

In the crystal structures, the bound oligosaccharides were confined at the binding sites in an extended conformation (Fig. 6A–C) and were well defined in the density maps (Fig. 5A, C, D). The oligosaccharides were bound to the binding sites with many water molecules: 29 molecules in NagB1/(GlcNAc)_2_, 28 molecules in NagB2/(GlcNAc)_2_, and 34 molecules in NagB2/(GlcNAc)_3_ (Fig. 6A–C). The polypeptide chains of NagB1 and NagB2 superimposed well (Fig. 5E), and both proteins recognized the bound oligosaccharide in a similar manner. The bound GlcNAc units were numbered starting at 1 from the non-reducing end. In both (GlcNAc)_2_ bound structures of NagB1 and NagB2 (Fig. 6A, B), the first GlcNAc interacted with the tryptophan residue Trp213 of NagB1 or Trp217 of NagB2 and formed hydrogen-bonding networks with surrounding residues. The O3, O4, O6, and O7 atoms of the first GlcNAc formed hydrogen bonds with Arg95, Asp401, and Ser330 of NagB1 and Arg96, Asp408, and Ser334 of NagB2. Other residues such as Asp91 and Glu167 of NagB1 and Asp92 and Glu168 of NagB2 interacted with GlcNAc mediated by water molecules. In NagB2/(GlcNAc)_2_, Lys416 interacted with the first GlcNAc via a water molecule. In contrast, Lys416 was not conserved in NagB1 (Fig. S1). The residue was substituted with Arg409, and the residue space was filled with Arg409 and Phe96 in NagB1. The second GlcNAc unit formed a CH/π interaction with Trp292 of NagB1 or Trp296 of NagB2 (Fig. 6A, B). The O1, O6, O7, and N2 atoms of the second GlcNAc formed hydrogen bonds with Arg64, Asp92, and Asn331 of NagB1 or Arg373, Asp92, and Asn335 of NagB2. Thr55 of NagB1 and Ile56, Asn57, and Gly165 of NagB2 interacted with the second GlcNAc through water molecules. Other residues, such as Phe115 and Phe165 of NagB1 and Phe116 and Phe166 of NagB2, hydrophobically interacted with (GlcNAc)_2_. In NagB1/(GlcNAc)_2_, Arg64 interacted with the second GlcNAc. In NagB2, this residue was substituted with Thr65 (Fig. S1), but the spatially closer Arg373 played an important role in recognizing the second GlcNAc. In the (GlcNAc)_3_ bound structure of NagB2 (Fig. 6C), the first and second GlcNAc units interacted with the proteins in a similar manner to the (GlcNAc)_2_ bound structures (Fig. 6A, B). However, the binding of the third GlcNAc moved the structural folds of two regions (Ser299–Asp305 and Gly365–Val374) outward, slightly widening the active-site groove, and causing the side-chain of Arg373 to shift in the opposite direction. The O1, O6, and N2 atoms of the third GlcNAc unit formed hydrogen bonds with Thr65, Asn57, and Trp296 of NagB2. The residues Arg59, Glu163, Gln280, Ser299, and Asp371 of NagB2 interacted with the third unit through water molecules (Fig. 6C).

The two domains formed similar closed ligand-binding structures in NagB1 and NagB2. These conformational changes occurred through rigid body rotation, similar to other SBPs (Sharff et al., 1992). In contrast, in the asymmetric unit of the ligand-free crystal of NagB2, the two polypeptide chains showed two different opening structures. The inter-domain rotation angle between the two chains was 27.1° and belonged to an open form (chain A) and a semi-closed form (chain B). When (GlcNAc)_2_ was bound, this angle changed to 56.8° (Fig. 6D) between the closed and open forms and 30.2° (Fig. 6E) between the closed and semi-closed forms. When (GlcNAc)_3_ was bound, the angles were 58.6° between the closed and open forms and 32.1° between the closed and semi-closed forms.

### Conservation of the a.a. Residues

**Fig. 7:**
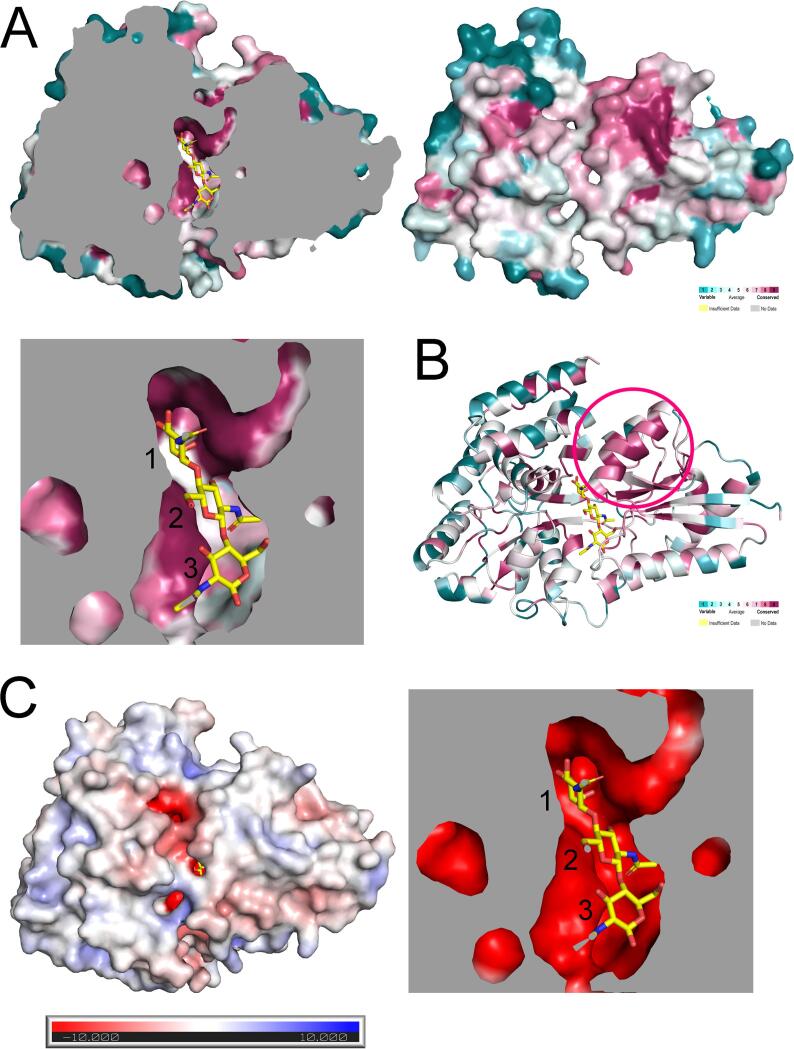
Amino acid conservation of NagB2 structure. (A) The surface of NagB2 with bound (GlcNAc)_3_ (yellow stick models) is colored with regions of the greatest variability (cyan), modest (white), and highest conservation (magenta) using the ConSurf server. The figure below is a close-up view of the binding site. The numbers are from the non-reducing end of the oligosaccharide. (B) The ribbon model was also shown to represent the conserved surface. The pink circle indicates the highly conserved alpha-helix region of domain II on the binding surface to the TMD. (C) The structure of NagB2/(GlcNAc)_3_ is also shown as electrostatic potentials at pH 7 (+10 to –10 kT/e). The figure on the right is a close-up view of the binding site. The numbers are from the non-reducing end of the oligosaccharide. (For interpretation of the references to colour in this figure legend, the reader is referred to the web version of this article.)

The conservation of the a.a. residues of both proteins was analyzed using the ConSurf server, based on the multiple sequence alignment with 150 a.a. sequences (Fig. 7A, B). The homologous sequences were automatically selected from the UniRef90 database (Suzek et al., 2015) using the server with CSI-BLAST (Angermüller et al., 2012). Almost all sequences (a.a. sequence identity at approximately 40–80%) belonged to uncharacterized bacterial proteins, mainly from gram-positive bacterial genera such as *Paenibacillus*, *Cohnella*, *Brevibacillus*, and *Geobacillus*. In the binding sites, the residues surrounding the first GlcNAc were highly conserved (Fig. 7A). However, the residues around the second and third units were less conserved than those around the first unit, which was similar to what was observed when comparing NagB1 and NagB2. On the other hand, the surface of the binding site was negatively charged regardless of a.a. conservation (Fig. 7C). In addition to the binding site, several surface regions and one alpha-helix in domain II in particular were highly conserved (Fig. 7B), suggesting that these regions play an important role in binding to other proteins, such as the transmembrane domains (TMDs) of ABC transporters.

## Discussion

*P*. str. FPU-7 degrades chitin by constitutively producing multiple chitinases (Itoh et al., 2013). In addition to these extracellular enzymes, ChiW induced by (GlcNAc)_2_ is expressed on the cell surface and hydrolyzes chitin to (GlcNAc)_2_ (Itoh et al., 2013, Itoh et al., 2016). *P*. str. FPU-7 has recently been found to hydrolyze (GlcNAc)_2_ to two GlcNAc residues in the cytosol via GlcNAcase PsNagA (Itoh et al., 2019). However, whether the disaccharides were transported across the *P*. str. FPU-7 membrane remained unclear. Many bacteria import oligosaccharides using ABC transporters, which are the most common class of sugar transporters in bacteria. SBPs, a component of ABC transporters, are responsible for trapping ligands that are imported through a specific pathway formed by two TMDs, and mainly define the specificity and affinity of transporters (Berntsson et al., 2010, ter Beek et al., 2014). SBPs that import (GlcNAc)_2_ have been found in gram-positive *S. coelicolor* A3(2) (Saito et al., 2007) and gram-negative *V. harveyi* (Hayes et al., 2017), and their various properties have been elucidated. In the present study, the *nagB1* gene, which was predicted to encode the SBP of sugar, was located next to the *nagA* (GlcNAcase) gene. However, no genes encoding the TMD of the ABC transporter were found in the vicinity of the *nagA* and *nagB1* genes. Instead, a *nagB1*-homolog, *nagB2*, with two adjacent TMDs was identified (Fig. 1A and B). These two TMDs had a.a. sequences with moderate similarity (approximately 20–33% identity) to those of trehalose (Liu et al., 2020), maltose (Oldham et al., 2007), and alginate ABC transporters (Maruyama et al., 2015), and were presumed to be components of a sugar ABC transporter. However, both NagB1 and NagB2 proteins showed a low a.a. sequence similarity with the previously known (GlcNAc)_2_ SBPs of *S. coelicolor* A3(2) and *V. harveyi* (a.a. sequence identity < 20%), making it difficult to identify both proteins as SBPs of (GlcNAc)_2_. Thus, we prepared and characterized both NagB1 and NagB2 proteins and confirmed that these proteins were SBPs of chitin oligosaccharides. We then analyzed their functions and structures, and clarified the SBP mechanism recognizing (GlcNAc)_2_.

Sequence analysis using the CSS-Palm server predicted that the NagB1 and NagB2 proteins were anchored on the cell membrane via post-translational modification by palmitic acid. Although these proteins specifically bound chitin-oligosaccharides such as (GlcNAc)_2_ and (GlcNAc)_3_ (Fig. 2), they recognized (GlcNAc)_2_ more tightly than (GlcNAc)_3_ (Table 2). On the cell surface, ChiW degrades chitin to disaccharides, and it is more convenient for disaccharides rather than trisaccharides to be recognized by both proteins. We confirmed that the NagB1 and NagB2 proteins were expressed in *P*. str. FPU-7 by gene expression and western blot analyses (Fig. 4). Both genes were expressed in the presence or absence of (GlcNAc)_2_. Although the NagB1- or NagB2-derived polyclonal antibodies could not distinguish between NagB1 and NagB2, at least one of the proteins was indeed expressed in the bacterium, suggesting that NagB1 or NagB2 helped to capture the oligosaccharides on the cell surface.

The recognition mechanism of chitin oligosaccharides was clarified by determining the crystal structures of these proteins. Both structures were similar and belonged to the SBP structural fold superfamily (Davidson et al., 2008, Rice et al., 2014, Scheepers et al., 2016). The SBP structural fold is classified into six clusters (A–F), and SBPs for sugar transport mainly belong to clusters B and D (Berntsson et al., 2010). Clusters B and D are further classified into subclusters B-I to B-V and D-I to D-IV (Scheepers et al., 2016). The structures of NagB1 and NagB2 were classified as subcluster D-I and contained two α/β domains connected by a hinge region consisting of three strands, with the substrate-binding pocket located between the two domains (Fig. 5). The binding sites of the oligosaccharides are large aqueous cavities that can accommodate up to three sugar residues, as revealed by the crystal structures. There seems to be no place for the fourth saccharide to bind, which is consistent with what would be expected from the DSF results with several saccharides. The kinetic parameters exhibited by both NagB1 and NagB2 for (GlcNAc)_2_ showed similar *K*_d_ values of approximately 200 nM. The a.a. residues for the recognition of (GlcNAc)_2_ on the binding site are also well conserved between both proteins. However, the a.a. residues around the third saccharide are less conserved. The difference in *K*_d_ values for (GlcNAc)_3_ is probably due to this divergence.

**Fig. 8:**
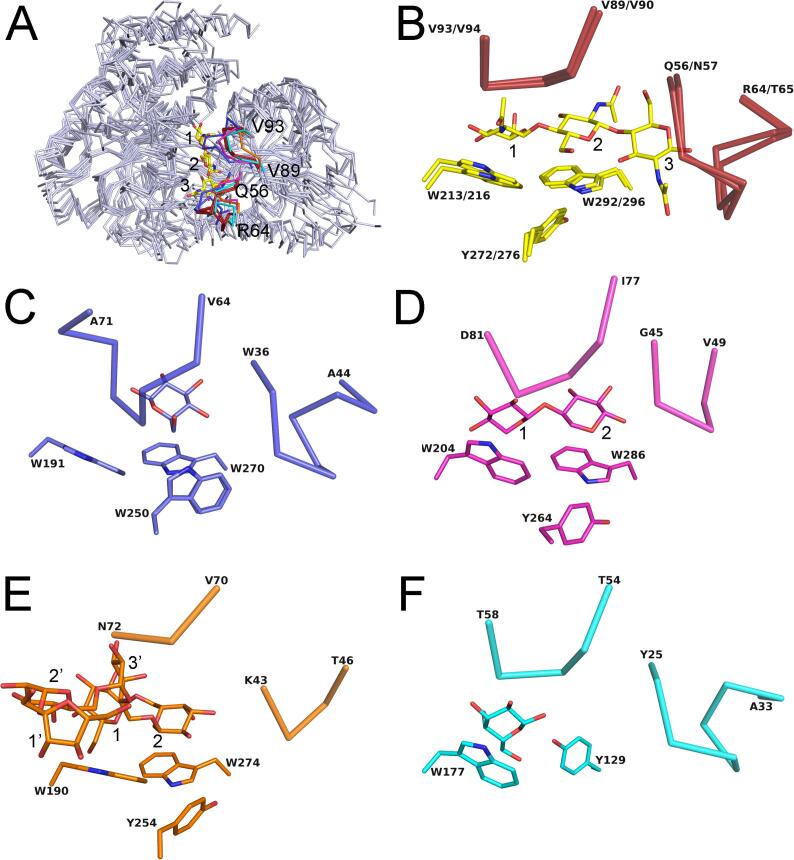
Structural comparisons of NagB1 and NagB2 with other SBPs. (A) The six overall structures (NagB1/(GlcNAc)_2_, NagB2/(GlcNAc)_3_, SBPs of glucose (PDB: 5DVI), xylooligosaccahrides (PDB: 3VXC), raffinose (PDB: 6PRE), and galactose (PDB: 3OO6)) were superimposed and represented by light blue ribbon models. The bound (GlcNAc)_3_ of NagB2/(GlcNAc)_3_ is represented by yellow stick models. Numbers 1, 2, and 3 indicate the number of saccharide locations from the non-reducing saccharide. The structures of the two loops, making the wall of the binding sites, are characteristic features of these proteins (NagB1 and NagB2 (red); SBP of glucose (blue); xylooligosacchrides (magenta); raffinose (orange); galactose (cyan)). (B) In the oligosaccharide binding sites of NagB1 and NagB2, Trp213, Tyr272, and Trps292 of NagB1 and Trp216, Tyr276, and Trp296 of NagB2 were well conserved in these proteins. The two loops (Gln56–Arg64 and Val89–Val93 of NagB1 and Asn57–Thr65 and Val90–Val94 of NagB2) are represented by red ribbons. The (GlcNAc)_3_ of NagB2/(GlcNAc)_3_ is represented by yellow stick models. (C) In the binding site of SBP of glucose, Trp250 is presented instead of Tyr. The two loops (Val64–Ala71 and Trp36–Ala44) are also represented by blue ribbon models. The bound Glc is represented by blue stick models. (D) In the binding site of SBP of xylooligosaccharides, Trp204, Tyr264, and Trp286 are present. The two loops (Ile77–Asp81 and Gly45–Val49) are also represented by magenta ribbon models. The bound xylobiose is represented by magenta stick models. (E) In the binding site of SBP of raffinose, Trp190, Tyr254, and Trp274 are present. The two loops (Val70–Asn72 and Lys43–Thr46) are also represented by orange ribbon models. The bound α-D-galactopyranose-(1–6)-α-D-galactopyranose-(1–6)-α-D-galactopyranose-(1–6)-[β-D-fructofuranose-(2–1)] α-D-glucopyranose is represented by orange stick models. (F) In the binding site of SBP of galactose, one tryptophan residue is not conserved, and tyrosine residue is presented instead. Another tyrosine is also absent in the structure. The two loops (Thr54–Thr58 and Tyr25–Ala33) are also represented by cyan ribbon models. The bound galactose is represented by cyan stick models. (For interpretation of the references to colour in this figure legend, the reader is referred to the web version of this article.)

Consistent with the results of the DALI search, the NagB1 and NagB2 structures were superimposed on SBPs of other sugars such as glucose, xylooligosaccharide, raffinose, and galactose (Fig. 8A). Although these SBPs have low a.a. sequence similarity (approximately 19–24% identity), two tryptophan residues such as Trp213 and Trp292 of NagB1 were well conserved in their binding sites (Fig. 8B-E), except for the SBP of galactose (Fig. 8F). In addition to the two tryptophan residues, one tyrosine residue (e.g., Tyr292 of NagB1) was also well conserved in the proteins (Fig. 8B, D, E), except for the SBPs of glucose and galactose (Fig. 8C, F). These residues belong to C-terminal domain II and form a platform for the sugar rings with CH/π interactions. Although the overall structures were very similar, the structures of the two loops of N-terminal domain I were different for each protein (e.g., Gln56–Arg64 and Val89–Val93 of NagB1), which resulted in different wall structures at the binding sites (Fig. 8). It was suggested that each protein forms a different wall structure and specifically recognizes hydroxy groups and other functional groups of sugars through this wall structure.

Upon binding to the ligand, SBP undergoes a conformational change from an open conformation to a closed conformation. This change is known as the “Venus flytrap” mechanism (Berntsson et al., 2010, Mao et al., 1982, Tirado-Lee et al., 2011, Vyas et al., 1988). The structure of NagB2 also changed significantly upon ligand binding, and the rotation angle was comparable to that of previously known structures (Fig. 6D). Although the structure of NagB1 in its ligand-free form has not yet been determined, considering the conservation of a.a. sequences and three-dimensional structures, it is likely that NagB1 undergoes the same structural changes as NagB2.

Although the genes *nagB1* and *nagB2* of SBPs for chitin oligosaccharides were constitutively expressed in the presence or absence of (GlcNAc)_2_, functions other than substrate binding are unknown. The neighboring genes of *nagB1* were predicted to encode a two-component regulatory system (histidine kinase and response regulator) (Fig. 1A). In contrast, the neighboring genes of *nagB2* were predicted to encode the TMD of the oligosaccharide ABC transporter (Fig. 1B). Some SBPs function as part of ABC transporters or two‐component regulatory systems (Scheepers et al., 2016). In *V. cholerae*, the SBP for (GlcNAc)_2_ functions not only as the SBP of the disaccharide-importing ABC transporter complex, but also as a component of the two-component regulatory (signal transduction) system together with a sensor histidine kinase and a response regulator that controls the expression of target genes such as chitinases (Li and Roseman, 2004). Thus, NagB1 may trap (GlcNAc)_2_ and induce the expression of chitinase genes, such as ChiW, via a phosphorylation relay in the system. Identifying the target genes that are induced is a challenge for the future. NagB2 may be involved in transporting (GlcNAc)_2_ across the membrane as a typical SBP of the ABC transporter with two predicted TMDs. However, there was no ATP-hydrolyzing enzyme (ATPase) in the nearby genes. In the genome of *Bacillus subtilis*, there are at least seven incomplete ABC sugar importers lacking genes encoding ATPases; MsmX at another locus has been proposed as a multitask ATPase, and the six incomplete transporters share this enzyme as a component (Leisico et al., 2020, Quentin et al., 1999). Similar multitask ATPases have also been found in streptococci (Marion et al., 2011, Tan et al., 2015). Thus, the chitin oligosaccharide uptake system (ABC transporter) of *P*. str. FPU-7 may utilize multitasked ATPase. On the other hand, although the two proteins may have different functions, they may complement each other's functions because NagB1 and NagB2 are similar in their a.a. sequences, conformations, and ligand binding abilities.

In conclusion, we identified and characterized *Paenibacillus* spp. NagB1 and NagB2 proteins as SBPs for chitin oligosaccharides. These findings will facilitate our understanding of the structural and functional aspects of chitin oligosaccharide transport and regulatory systems in bacteria.

## Funding

This work was supported in part by a Grant-in-Aid for Scientific Research (C) (TI, Grant Numbers 16 K08114 and 19 K06340) from the Japan Society for the Promotion of Science.

## CRediT authorship contribution statement

**Takafumi Itoh:** Conceptualization, Funding acquisition, Formal analysis, Investigation, Methodology, Project administration, Supervision, Validation, Visualization, Writing - original draft. **Misaki Yaguchi:** Investigation, Writing - review & editing. **Akari Nakaichi:** Investigation, Writing - review & editing. **Moe Yoda:** Investigation, Writing - review & editing. **Takao Hibi:** Investigation, Formal analysis, Supervision, Validation, Writing - review & editing. **Hisashi Kimoto:** Conceptualization, Resources, Supervision, Project administration, Writing - review & editing.

## Declaration of Competing Interest

The authors declare that they have no known competing financial interests or personal relationships that could have appeared to influence the work reported in this paper.
