# Supplementary material for: Structural characterization of two solute-binding proteins for N,N′-diacetylchitobiose/N,N′,N′′-triacetylchitotoriose of the gram-positive bacterium, Paenibacillus sp. str. FPU-7
Source: J Struct Biol X. 2021 Jun 10;5:100049. doi: 10.1016/j.yjsbx.2021.100049 (PMC8233162; doi:10.1016/j.yjsbx.2021.100049)
Supplement: Supplementary data 1 [file mmc1.pdf]

Supplementary Information

Structural characterization of two solute-binding proteins for *N*,*N*'-diacetylchitobiose/*N*,*N*',*N*''-triacetylchitotoriose of the gram-positive bacterium, *Paenibacillus* sp. str. FPU-7.

Takafumi Itoh[a*], Misaki Yaguchi[a], Akari Nakaichi[a], Moe Yoda[a], Takao Hibi[a], and Hisashi Kimoto[a*]

[a]Department of Bioscience and Biotechnology, Fukui Prefectural University, 4-1-1 Matsuokakenjyoujima, Eiheiji-cho, Yoshida-gun, Fukui 910-1195, Japan

***To whom correspondence should be addressed**: Takafumi Itoh and Hisashi Kimoto, Department of Bioscience and Biotechnology, Fukui Prefectural University, 4-1-1 Matsuokakenjyoujima, Eiheiji-cho, Yoshida-gun, Fukui 910-1195, Japan; Phone +81-776-61-6000; Fax +81-776-61-6015; E-mail ito-t@fpu.ac.jp, kimoto@fpu.ac.jp

Table S1. Oligonucleotide primers used in cloning and RT-PCR.

Primer name	Primer sequence (5´ to 3´)
Cloning	
PsNagB1_49T-F	GGAGATATA**CATATG**ACGGTATCGCTGCGGCATAC
PsNagB1-R	GTGGTGGTG**CTCGAG**TTTTTTCATATCGCGGTTGG
PsNagB2_21G-F	GGAGATATA**CATATG**GGTTGCGGCGGTACAGCA
PsNagB2-R	GTGGTGGTG**CTCGAG**CTTGCCGGCGTTTGCTTC
qPCR	
NagB1_qRT_F	CGGTACGATACGCTGCTGAA
NagB1_qRT_R	CCAGCTTCCGTCGAACATCA
NagB2_qRT_F	ATCTTCAACCTCTTCGGCGG
NagB2_qRT_R	TACCGTCAACCGTGAACTCG
PsFPU7_16SrRNA-F	ATGCGTAGCCGACCTGAGA
PsFPU7_16SrRNA-R	GCGTTCTTCCTTGGCAACAG

Bold characters indicate recognition site of restriction enzyme, *Nde*I or *Xho*I.

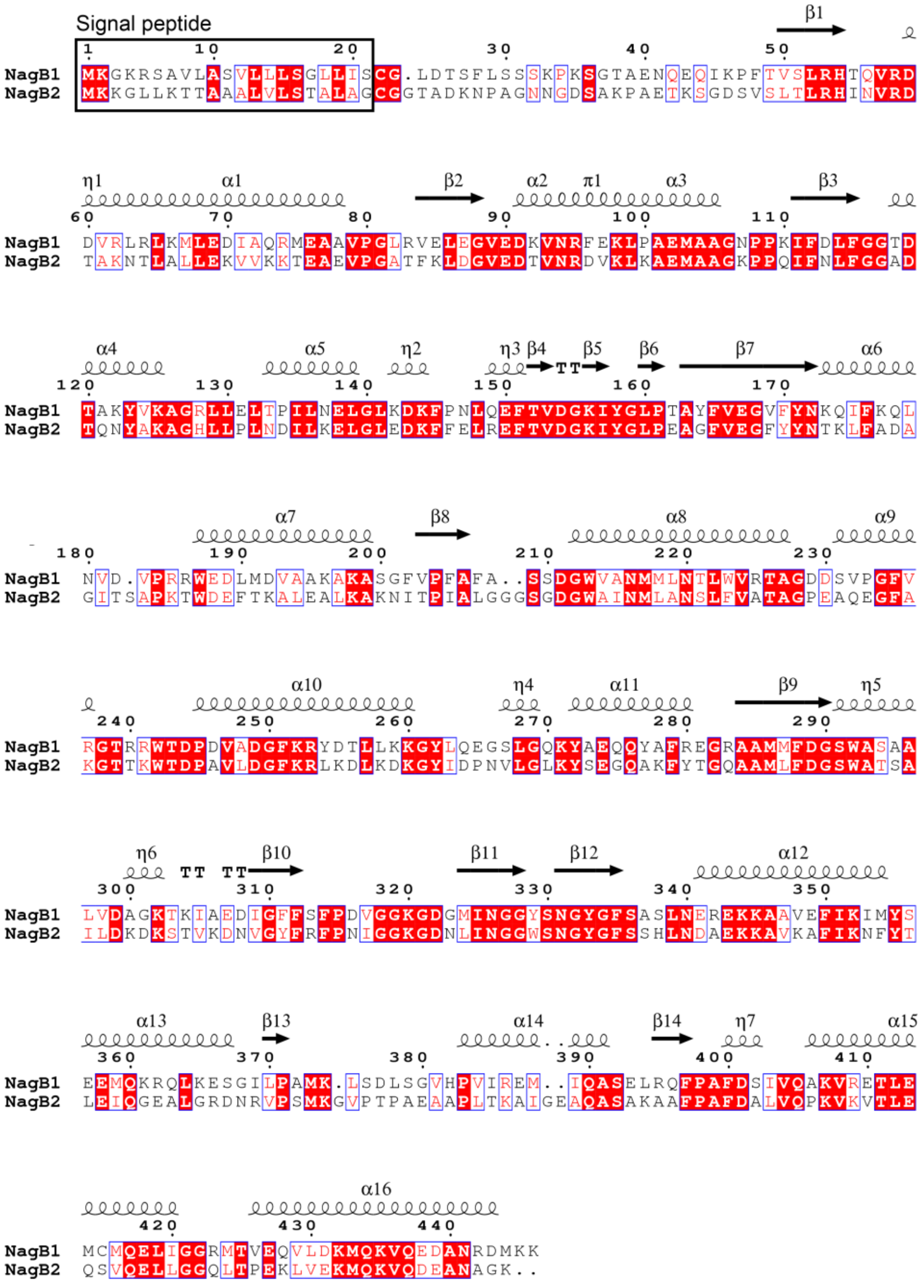

Fig. S1. Amino acid sequence alignment of NagB1 and NagB2.

The first line represents secondary structure assignment of NagB1/$(GlcNAc)_2$ according to the program DSSP. The a.a. sequences of NagB1 and NagB2 were aligned by ClustalW program. The residue numbers of NagB1 are displayed above the sequence. The predicted signal peptide sequences (Met1-Ser21 of NagB1 and Met1-Gly21 of NagB2) are boxed in black. The figure was generated by using ESPript 3.0 (https://espript.ibcp.fr/ESPript/ESPript/).
